# The relationship between ignition and oxidation of molten magnesium alloys during the cooling process

**DOI:** 10.3389/fchem.2022.980860

**Published:** 2022-08-11

**Authors:** Xinyi Zhao, Tao Gu, Haiyang Zhang, Zeyu Wang, Hassaan Ahmad Butt, Yucheng Lei

**Affiliations:** ^1^ School of Materials Science and Engineering, Jiangsu University, Zhenjiang, China; ^2^ Skolkovo Institute of Science and Technology, Moscow, Russia

**Keywords:** magnesium alloys, ignition, oxidation, casting process parameters, pouring temperature, cooling rate

## Abstract

Ignition of magnesium alloys during casting processes limits their processability and applications. For identifying the ignition mechanism of magnesium alloys during solidification, a Mg-Al-Zn alloy was solidified with different cooling rates and pouring temperatures. The oxide scale morphologies and thicknesses were identified by SEM and energy dispersive spectrometer. Based on the experimental results, the oxidation kinetics and heat released were calculated and the relationship between oxidation and ignition was discussed in detail. The calculation results indicate that oxide rupture directly induces combustion of the melt. The rupture route of the oxide scale was determined to be buckling cracks according to the experimental and calculation results. Based on the buckling mechanism of the oxide scale, the ignition criterion during solidification was correlated to the pouring temperature, cooling rate and casting modulus. This work reveals the underlying relationship between ignition and casting process parameters, and it helps to develop new technology for inhibiting ignition of molten magnesium alloys.

## 1 Introduction

Magnesium alloys are commonly recognized as one of the most potential materials in the aerospace industry and biomedicals (Mehra et al., 2018; Yao et al., 2018; Cao et al., 2021). Casting is the main manufacturing process for magnesium alloy components. However, molten magnesium and its alloys are highly reactive in an oxidizing atmosphere and are easily ignited during the melting and casting processes, which extremely limits their processing and applications. Many investigations have focused on the development of ignition-proof magnesium alloys and protective atmospheres. According to recent research, Ca (Inoue et al., 2017), CaO (Lee 2016), Be (Tan et al., 2018), Sr (Villegas-Armenta and Pekguleryuz, 2020), and several rare Earth elements (Gd, Nd, Y, et.) (Tan et al., 2019; Cai et al., 2021; Wu et al., 2021) may inhibit the ignition of magnesium alloys by forming a continuous protective oxide layer on the surface. However, such solutions cannot provide long-term protection. Even with the addition of these reactive elements, protective atmospheres are still needed in the melting and casting processes of magnesium alloys. The most commonly used protective atmosphere in industry is sulphur hexafluoride (SF_6_), due to its non-toxic, non-flammable properties and excellent protection performance (Emami et al., 2014). Unfortunately, SF_6_ has a strong negative greenhouse effect and a long atmospheric lifetime, with its global warming potential (GWP) being 23,900 and an atmospheric lifetime of 3,200 years. With increasing environmental concerns in recent years, the use of SF_6_ in industries is strictly restricted or forbidden in many countries throughout the world. Several alternative gases have been found so far. Hexafluoropropylene (HFP) has been suggested as the protective gas for molten magnesium alloys (Chen and Jiang 2021). Moreover, Ha and Kim (Ha and Kim 2006) investigated the melt protection properties of inhibitors (SF_6_, HFC-134a and SO_2_)/air cover gas mixtures for different magnesium alloys. The results indicated that HFC-134a and SF_6_ showed better protection properties than SO_2_. However, the study of such gases requires more thorough testing and demonstration since studies tend to represent perfect environmental conditions, whereas industry environments are more complex and dynamic.

The term of ignition temperature is commonly used to describe a susceptibility of magnesium and its alloys to initiate burning during heating (Han et al., 2020). It should be noticed that ignition temperature is not an intrinsic property of magnesium alloys and it is significantly influenced by the heating conditions. Generally, magnesium alloys ignite at the temperatures near their melting points in a continuous heating process and the ignition temperature varies with heating rate (Han et al., 2020). Moreover, magnesium alloys may ignite at temperatures extremely below their liquidus in isothermal holding process. With the prolonging of isothermal holding time, the ignition temperature of AZ91D alloy is reduced from 650°C, representing the liquid state, to as low as 520°C, representing mainly the solid state with liquid pools (Mebarki, et al., 2005). For most of casting magnesium alloys, the ignition temperature could be as low as 427°C in a prolonged heating process in air (Czerwinski 2014). For revealing the underlying mechanism of ignition, many researchers have tried to observe the oxidation processes of magnesium alloy melts in various conditions (Zhao et al., 2019). It has been believed that the ignition of magnesium alloy melts is closely related to the rupture of surface protective oxide scales. In this process, magnesium vapor overflows from the melts causing vigorous oxidation and thus releasing a large amount of heat, leading to the combustion of the melts (Tekumalla and Gupta 2017). This process is nearly impossible to be directly observed and the critical conditions required for oxide scale rupture remains unknown. Although problematic, attaining the critical conditions of surface oxide scale rupture is essential for the control of ignition. During casting processes, the cooling melts in the mold could react with the in-mold atmosphere and cause combustion of the castings. This is overlooked in most publications pertaining to oxidation and ignition, where the focus remains on heating conditions and isothermal holding processes. The ignition behaviors of magnesium melts in the cooling processes have not been well studied and remain an area which requires scientific attention.

This work aims to reveal the critical thermal conditions for the ignition of magnesium melts in the cooling processes, based on experimental analysis and theoretical calculations. AZ31 melt was selected to solidify under different cooling rates. The compositions and thicknesses of oxide scales formed on AZ31 surface were analyzed by SEM and EDS and the rupture morphologies of the oxide scales were observed. Based on the experimental results and thermodynamic and kinetic analysis, the oxidation kinetics of AZ31 in cooling processes was elaborated upon. On this basis, the oxide rupture critical conditions were estimated.

## 2 Materials and methods

### 2.1 Materials

**FIGURE 1:**
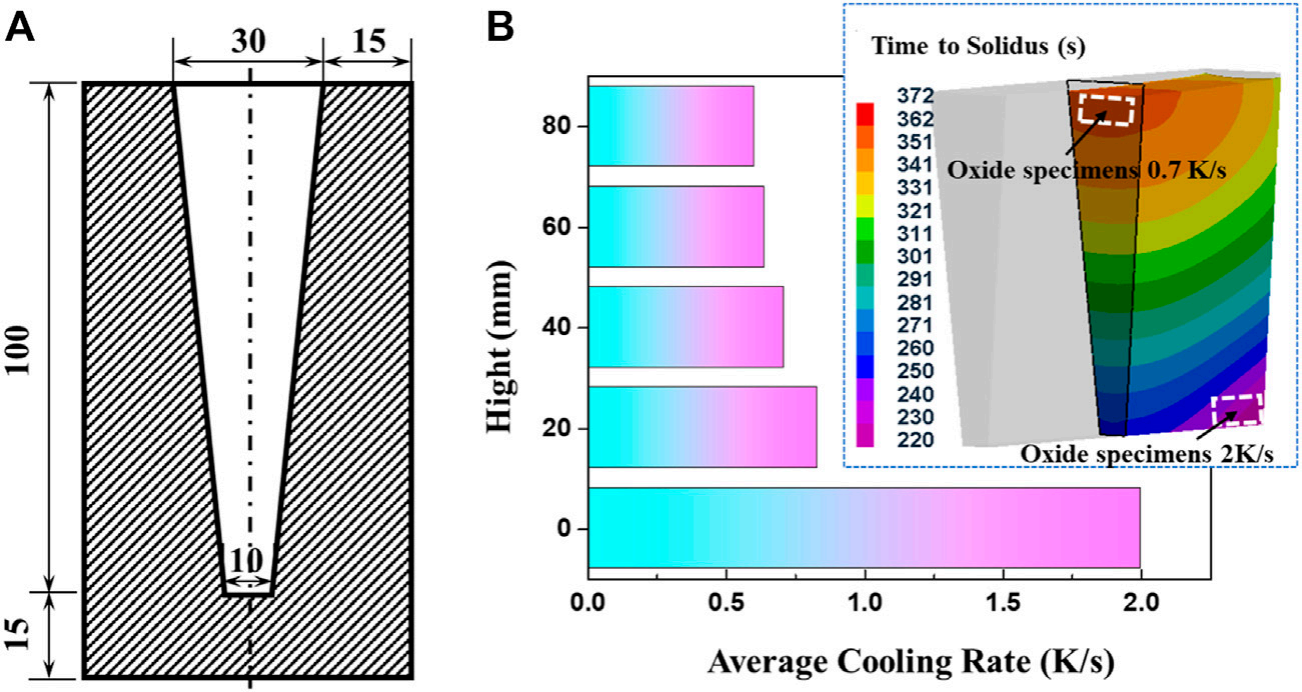
The structure of resin sand mold **(A)** and the cooling rates at different sections **(B)**.

The AZ31 alloy was melted in a mild steel crucible using an electrical resistance furnace. The starting materials were 99.9% pure Mg, Mg-30wt.%Al and Mg-15wt.%Zn master alloys. The pure Mg was melted, followed by the addition of Mg-Al and Mg-Zn master alloys after it was heated to 973 K. The mixed melt was held at 1023 K for 20 min and then poured into a resin sand mold with Y-type wedge. The whole melting process was protected by RJ2 cover flux (main compositions: 38–46% MgCl_2_, 32–40% KCl, 5–8% BaCl_2_, 3–5% CaF_2_, and 8% NaCl + CaCl_2_), and the pouring process was protected by a gas mixture of CO_2_/SF_6_. The structure of the resin sand mold used in this work is shown in Figure 1. The corresponding cooling rate was estimated to be ∼2 K/s and was ascertained through ProCAST software. The cooling rate used in this work is the average cooling rate during the whole solidification process. It is estimated with the pouring temperature, solidus temperature and solidification time. In order to achieve a low cooling rate, several melts were cooled from 993 K, 1033 K, and 1073 K to solidus within the furnace. The temperature during the solidification process was tested by a thermocouple in the melt and the cooling rate was seen to be ∼0.3 K/s. Inductively Coupled Plasma-Atomic Emission Spectroscopy (ICP-AES) measurements indicated the composition of the alloy investigated was Mg-2.82Al-0.75Zn (wt%).

### 2.2 Methods

The oxide specimens formed at cooling rates of 0.7 K/s and 2 K/s were cut from the wedge castings at different sections, as shown in Figure 1B. The oxide specimens formed at 0.3 K/s were manufactured from the top surface of castings solidified in crucible. The oxide specimens for cross-section observation were sequentially polished by SiC papers of #500, #1200, #2000 and 1 μm diamond paste. The oxide specimens for surface analysis were directly employed after washing by alcohol. The morphologies and thicknesses of the surface oxide scales were observed by A ZEISS ΣIGMA field emission scanning electron microscope (FE-SEM) equipped with energy dispersive spectrometer (EDS). The oxide scales formed on the castings solidified within the furnace were also analyzed. More than five typical regions for each specimen were subjected to the oxide thickness measurements and the average values were employed in the following discussion and calculations.

## 3 Results and discussion

### 3.1 Oxidation of AZ31 in cooling processes

**FIGURE 2:**
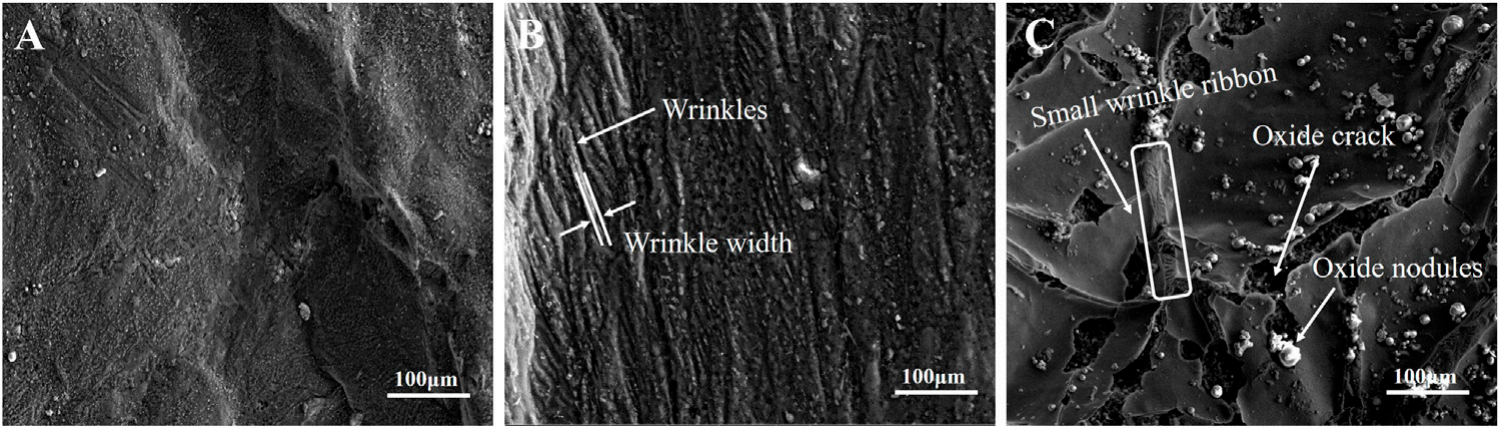
The surface morphologies of oxide scale formed on the castings.

The morphologies of the surface oxide scale formed on the castings under different cooling rates can been seen in Figure 2. When the cooling rate is 2 K/s, the surface oxide scale remains dense and continuous. Neither oxide cracks nor wrinkles are observed, as shown in Figure 2A. However, oxide wrinkles almost cover the entire surface when the cooling rate of the melt decreased to 0.7 K/s. According to the SEM results shown in Figure 2B, the average width of the oxide wrinkles is estimated to be ∼10 μm. When the cooling rate is reduced to 0.3 K/s, oxide cracks and nodules can be seen on the surface, as shown in Figure 2C, indicating ignition taking place. It can be found that the thickness of the oxide scale significantly increases compared to those formed under higher cooling rates, seen when observing the fracture morphologies of the oxide cracks. Moreover, the number of oxide wrinkles obviously decreases, when the cooling rate is lowered from 0.7 K/s to 0.3 K/s. Cracks mainly appear on the oxide wrinkle ridges and several wrinkle sections are filled with continuous small wrinkle ribbons, as marked in Figure 2C. The morphologies of the wrinkle ribbon are similar to the wrinkles formed at a cooling rate of 0.7 K/s. This phenomenon indicates that the oxide scales tend to rupture at the wrinkle ridges as the thickness is increased and the cracks can be partly recovered by newly formed oxide scale, which prefers to appear with small size wrinkles due to its thin thickness.

**FIGURE 3:**
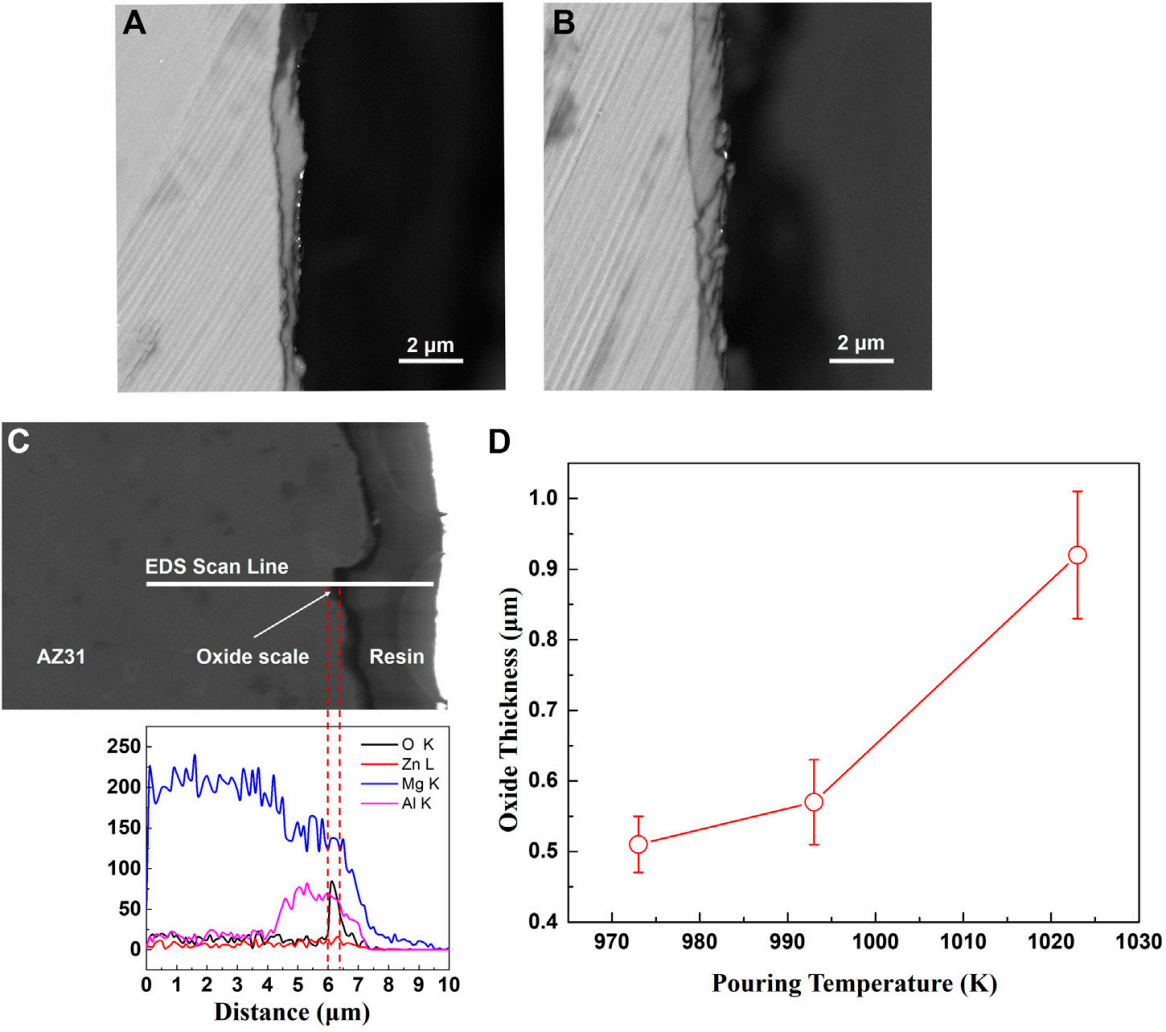
The cross-sections of oxide scales formed on the castings. **(A)** cooling rate 0.7 K/s **(B)** cooling rate 0.3 K/s **(C)** EDS analysis of oxide scale **(D)** the thickness of oxide formed at a cooling rate of 0.3 K/s.

The cross-section morphologies of the oxide formed at cooling rates of 0.7 K/s and 0.3 K/s are shown in Figure 3. The oxide scale formed at 2 K/s is too thin to be observed by SEM. According to Figures 3A,B, even though there are wrinkles and cracks appearing on the surfaces of the specimens, the oxide remains continuous and tightly adherent with the substrate metal. It can be inferred that the uncracked oxide scale remains protective to the melt. The oxide composition is identified to be MgO using EDS line scanning, as shown in Figure 3C, and the corresponding oxide thickness can be determined by coupling the distribution of oxygen element and the oxide cross-section morphology. The analysis of the oxide thickness formed at a cooling rate of 0.3 K/s and different pouring temperatures are summarized in Figure 3D. The average oxide thickness significantly increases with pouring temperature, approximately following an exponential law.

**FIGURE 4:**
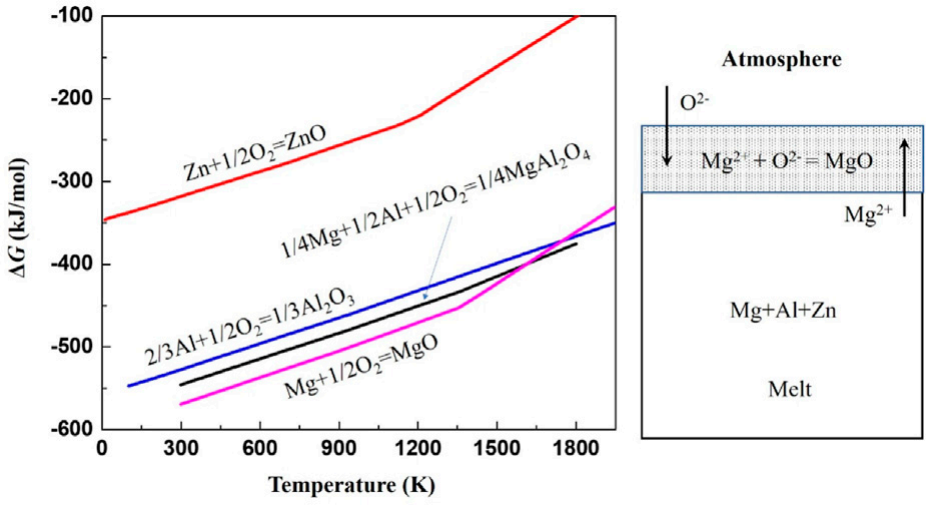
The oxidation reaction at the interface of melt/atmosphere and the Gibbus free energies of the formation of oxides.

According to the free energies of the formation of oxides as shown in Figure 4, only MgO could stably form at the melt surface. The thermodynamics analysis agrees well with the experimental results. Therefore, the following discussion about the oxidation and ignition mainly focused on the MgO scale.

### 3.2 Kinetics of oxidation during solidification

According to the experimental results mentioned above, the oxide scale formed on the surface of AZ31 melt is mainly MgO. As the growth of MgO is controlled by the diffusion of Mg^2+^, the kinetics of isothermal oxidation of the AZ31 melt can be estimated based on Fick’s first law, like most of the diffusion-controlled corrosion processes (Yan et al., 2022). Corrosion behavior of stainless steel-tungsten carbide joints brazed with AgCuX (X = In, Ti) alloys. Corrosion Science, 200, 110,231.). The relationship between diffusion rate per unit area and the concentration gradient is as follows:

$$J=-D\frac{C_{Mg^{2+}}-C_{Mg^{2+}}^{0}}{x}$$ (1)

**FIGURE 5:**
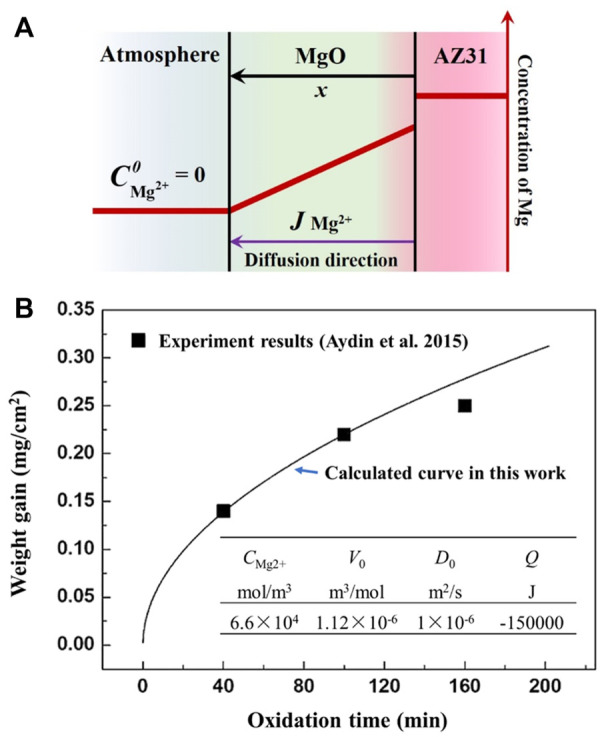
Illustration of the oxide growth model and the corresponding calculation result. **(A)** The kinetics calculation model used in this study **(B)** the calculated result of oxide growth on magnesium at 773 K.

Where *J* is the flux of Mg^2+^ in the direction of the *x*-axis, *D* is the diffusivity coefficient of Mg^2+^ in MgO, $C_{Mg^{2+}}^{0}$ and $C_{Mg^{2+}}$ represent the concentration of Mg^2+^ at the interface of the atmosphere/oxide and oxide/melt, respectively. It is assumed that the Mg^2+^ at the interface of atmosphere/oxide can immediately react with oxygen and form MgO. Therefore, the concentration of Mg^2+^ at the interface of atmosphere/oxide is 0, as seen in Figure 5A. At the beginning of the oxidation process, the new MgO scale is uniform and dense and the effects of defects in MgO on the Mg^2+^ diffusion is neglected. Hence, the thickness of the MgO scale can be expressed as follow:

$$\frac{dx}{dt}=V_{0}J=\frac{-DV_{0}C_{Mg^{2+}}}{x}$$ (2)

Where *V*_0_ is the molar volume of MgO and the diffusion coefficient *D* is not constant as its value depends on temperature:

$$D=D_{0}\exp\left(-\frac{Q}{RT}\right)$$ (3)

The thickness of MgO can be obtained by Eq. 4, come to by integrating Eq. 3:

$$x^{2}=-2D_{0}\exp\left(-\frac{Q}{RT}\right)V_{0}C_{Mg^{2+}}t$$ (4)

Based on Eq. 4, the weight gain of the oxidation process at 773 K is estimated as shown in Figure 5B. For the isothermal oxidation processes, the relationship between oxide scale thickness and oxidation time follows a parabolic growth law.

It can be seen in Figure 5B that although the influence of grain boundary and micro-defects is ignored, the calculated results are in good agreement with the experimental results of Aydin et al. (Aydin et al., 2015) within 100 min. The deviation after a long-time of oxidation (more than 120 min) may be caused by the structural transformation of the oxide scale in the actual oxidation process. In addition, because only the diffusion factor is considered, the calculation model can only analyze the oxidation process when there is a protective oxide layer present on the surface. As the oxidation processes during melt solidification definitely can not last for more than 100 min, it is reliable to estimate the oxide growth during solidification through the diffusion model mentioned above.

During the solidification process, the diffusion coefficient of Mg^2+^ would change its value with temperature. Therefore, the oxide growth described by Eq. 4 changes and follows Eq. 5:

$$\int xdx=-V_{0}\Delta C_{Mg^{2+}}D_{0}\int \exp\left[\frac{Q}{RT\left(t\right)}\right]dt$$ (5)

Where *T*(*t*) is the temperature of melt, which is a function of time *t*. Assuming that, there is a linear relationship between the melt temperature and time, and the pouring temperature is *T*_*0*_ (K), the cooling rate of melt is *a* (K/s). Equation 5 can be rewritten as:

$$\frac{1}{2}x^{2}=K\int_{0}^{t}\exp\left[\frac{Q}{R\left(T_{0}-at\right)}\right]dt$$ (6)

**FIGURE 6:**
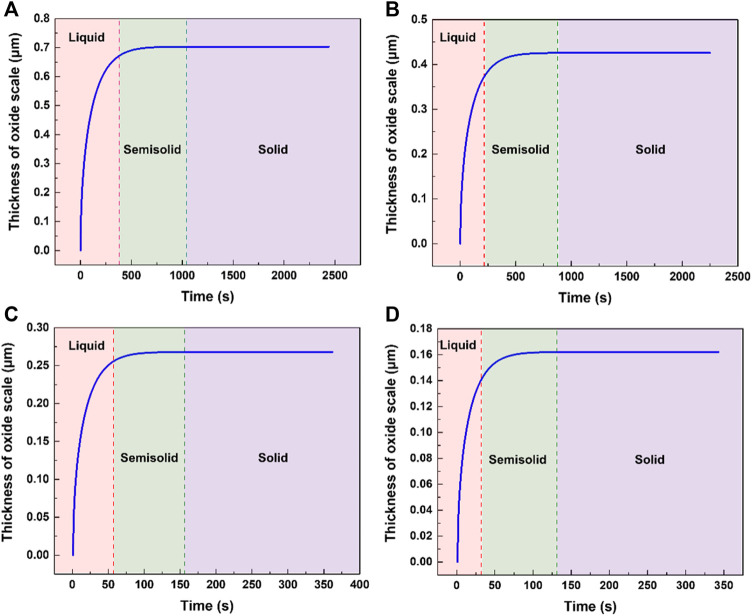
The calculation results of oxide thickness formed under different conditions. **(A)** pouring temperature 1023 K, cooling rate 0.3 K/s. **(B)** pouring temperature 973 K, cooling rate 0.3 K/s. **(C)** pouring temperature 1023 K, cooling rate 2 K/s. **(D)** pouring temperature 973 K, cooling rate 2 K/s.

Where *K* is a constant for $-V_{0}\Delta C_{Mg^{2+}}D_{0}$ . However, the integral result of Eq. 6 is not an elementary function, and there is no analytical solution for it. Liu et al. obtained an approximate solution by KAS and FWO methods (Liu et al., 2015). Based on that, the oxide growth thickness during solidification can be calculated as shown in Figure 6.

**FIGURE 7:**
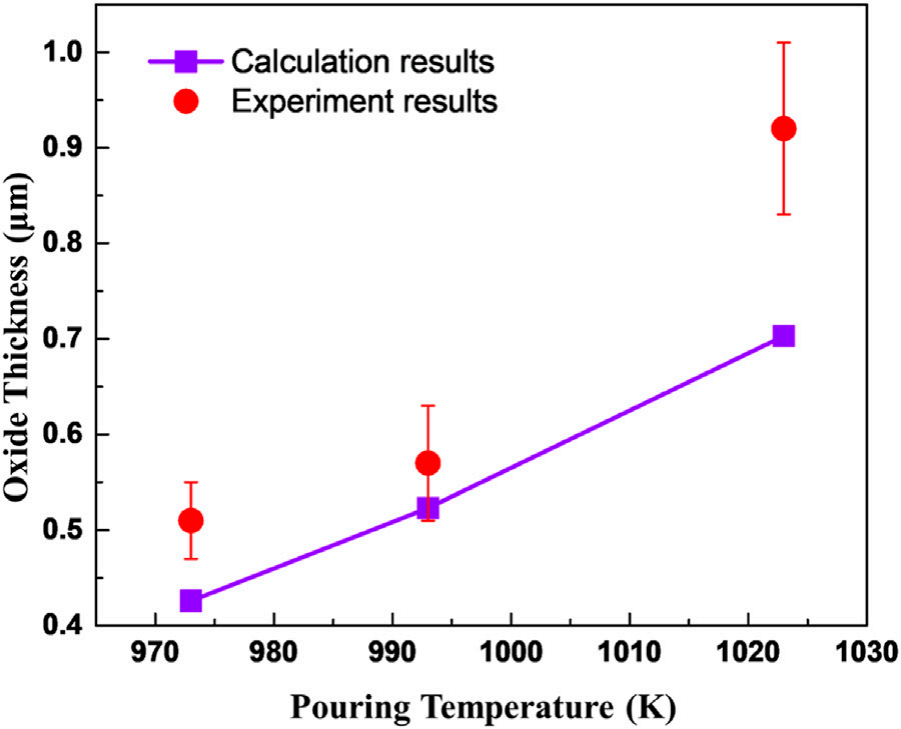
The thicknesses of oxide obtained through experiments and calculation.

It can be seen that the oxide mainly grows during the liquid cooling stage of AZ31. When the temperature of the metal is below its liquidus, the oxide thickness changes to a lesser degree. The experimental and calculated results meet well as shown in Figure 7. The oxide thicknesses obtained through experiments of high pouring temperatures are slightly larger than the calculated results and this may be attributed to the cracking of the oxide scale permitting a faster oxidation rate than the diffusion-controlled oxidation.

### 3.3 Effect of heat releasing of oxidation on the cooling of AZ31 melts

#### 3.3.1 The thermal effects of stable oxidation

During casting processes, the heats of melt solidification and surface oxidation reaction are all released through heat conduction of the casting mold. Flemings et al. developed an analytical expression to describe the temperature field of mold (Flemings 1974),

$$T=T_{S}+\left(T_{S}-T_{0}\right)\mathrm{erf}\left(\frac{x}{2\sqrt{at}}\right)$$ (7)

Where *T*_*s*_ is the temperature at the interface of mold/casting, *T*_*0*_ is the temperature at the outer surface of the mold, *x* is the distance to the mold inter-surface, *a* is the heat conduction rate of mold and *t* represents time.

The heat flux intensity at the interface of casting/mold is related to the instantaneous temperature gradient in the mold. Thus, it can be described as,

$$q=-\lambda \left(\frac{\partial T}{\partial x}\right)$$ (8)

Combined Eq. 7 and 8 can be rewritten as,

$$q=\sqrt{\frac{\lambda \rho c}{\pi t}}\left(T_{S}-T_{0}\right)$$ (9)

Where *λ*, *ρ,* and *c* are all physical parameters of mold, representing heat conduction coefficient, density, and thermal capacity, respectively.

As the ignition temperatures of magnesium alloys are mostly above their solidus temperatures, only the heat exchange during the liquid melt cooling process is considered. Ignoring the thermal capacity change of magnesium in the liquid state, there is a linear relationship between the melt temperature and the heat released. The heat released per mole of melt with its temperature decreasing by 1 K is *ΔH*_*S*_ (34 J/mol K, obtained by thermodynamic calculation) and thus the heat release during the cooling process of casting can be taken as:

$$q=\Delta H_{S}\frac{\rho_{L}h}{m}\frac{\partial T}{\partial t}$$ (10)

Where *ρ*_*L*_ is the density of magnesium alloy, *h* is half of the casting thickness and *m* is the molar mass of melt. The heat released by the melt is considered to be equal to the thermal conductivity of the mold. Combining Eqss 9, 10, the relationship between the casting temperature and cooling time is obtained as follow:

$$\Delta H_{S}\frac{\rho_{L}h}{m}\ln\left(\frac{T_{P}-T_{0}}{T_{S}-T_{0}}\right)=2\sqrt{\frac{\lambda \rho c}{\pi }}\sqrt{t}$$ (11)

Where *T*_*S*_ is the temperature of casting after cooling time *t* and *T*_*P*_ is the pouring temperature.

During the solidification process, the growth of the surface oxide scale is due to the interface reaction of the melt and mold. Considering the heat released by oxidation of the melt, Eq. 10 changes to:

$$q=\Delta H_{S}\frac{\rho_{L}h}{m}\frac{\partial T}{\partial t}+\frac{\Delta H_{O}\Delta C_{Mg^{2+}}D_{0}}{x}\exp\left(\frac{-Q}{RT}\right)$$ (12)

Where Δ*H*_*O*_ is the molar heat release of the oxidation reaction. The heat of oxidation still needs to be conducted through the mold. The effects of heat release during oxidation on the temperature of the casting can be expressed by combining Eqs. 9, 12:

$$\sqrt{\frac{\lambda \rho c}{\pi t}}\left(T-T_{0}\right)=\Delta H_{S}\frac{\rho_{L}h}{m}\frac{\partial T}{\partial t}+\frac{\Delta H_{O}\Delta C_{Mg}D_{0}}{x}\exp\left(\frac{-Q}{RT}\right)$$ (13)

Assuming that the surface oxide scale is formed after filling, and the thickness of the oxide scale is approximately considered as:

$$x=\sqrt{2DV_{0}\Delta C_{Mg^{2+}}t}$$ (14)

The temperature of the casting with thermal effects of oxidation can be obtained by substituting Eq. 14 into Eq. 13 and integrating:

$$\Delta H_{S}\frac{\rho_{L}h}{m}\ln\left(\frac{T_{P}-T_{0}}{T_{S}-T_{0}}\right)=\left(2\sqrt{\frac{\lambda \rho c}{\pi }}-\frac{\Delta H_{O}}{T_{P}-T_{0}}\sqrt{\frac{2\Delta C_{Mg}D}{V_{0}}}\right)\sqrt{t}$$ (15)

**FIGURE 8:**
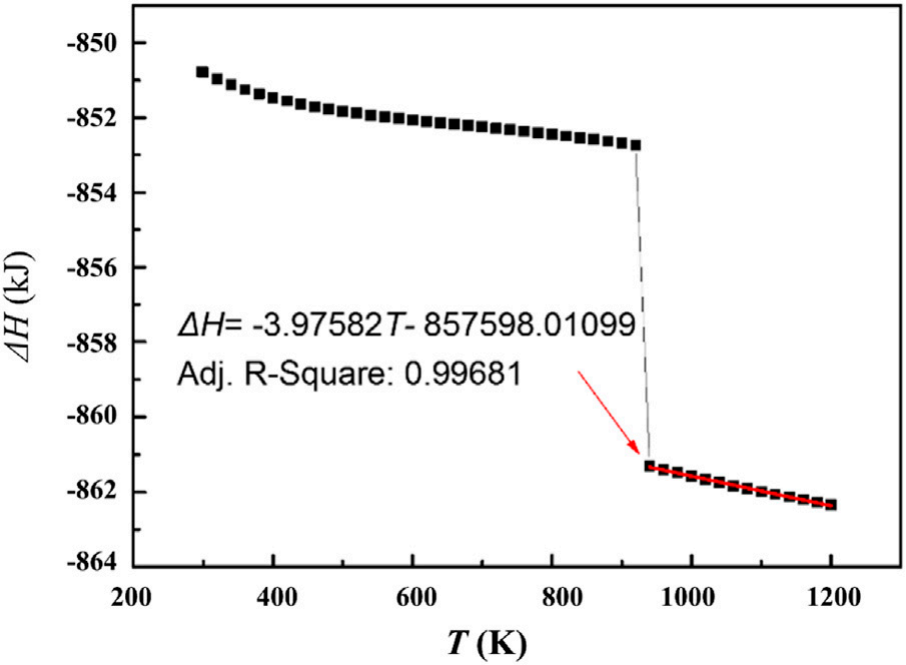
The heat release of magnesium oxidation as a function of temperature.

Based on the database SSUB6 of Thermo-Calc, the molar heat release of the oxidation reaction is calculated as shown in Figure 8. For liquid magnesium, the heat release can be characterized well by a linear function of temperature. Therefore, the Δ*H*_*O*_ in Eq. 15 is also a function of temperature (and time). In order to simplify the calculation, the molar heat release of the oxidation reaction in the process of cooling from temperature *T*_*0*_ to *T*, can be expressed by the molar heat release values at *T*_*0*_ and *T*:

$$\Delta H_{O}\left(T\right)=\frac{\Delta H\left(T_{0}\right)+\Delta H\left(T\right)}{2}=\Delta H\left(\frac{T_{0}+T}{2}\right)$$ (16)

Substituting Eq. 16 into Eq. 15, the exothermal effects of stable oxidation during magnesium melt cooling can be estimated.

**FIGURE 9:**
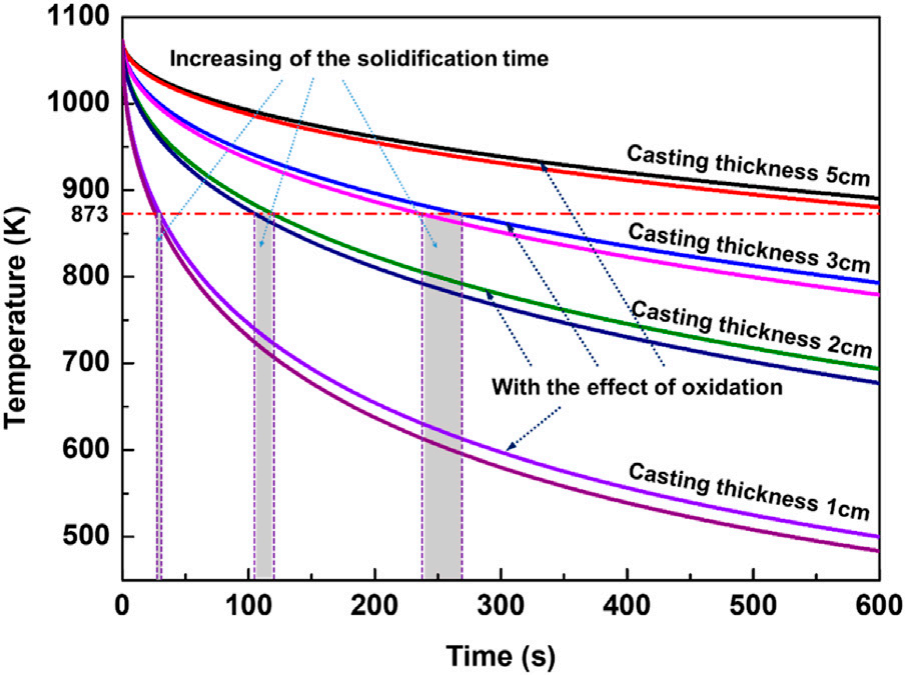
Effects of exothermic oxidation on the temperature of castings.

According to the experimental results, AZ31 melt will ignite when its pouring temperature reaches 1073 K and the solidification time exceeds 10 min. Therefore, the heat released by stable oxidation with a pouring temperature of 1073 K was calculated, and the results are shown in Figure 9. The cooling curve of the castings with the consideration of exothermic oxidation is very close to that without any oxidation effect. With the increase of casting wall thickness, the two temperature curves are closer, but their difference of solidification time increases. As shown in Figure 9, under the same solidification temperature (873 K), the increasing of solidification time associated with wall thickness of 3 cm, 2 cm, and 1 cm caused by oxidation is ∼31 s, 15 s, and ∼4 s, respectively, which indicates that the oxidation process increases the solidification time more for larger wall thickness castings.

In general, the temperature curves of magnesium alloy melt show a decreasing trend with time when considering the influence of oxidation process. It illustrates that if the growth of oxide layer on the surface of the magnesium alloy is always controlled by diffusion, the heat released by this stable oxidation reaction is not enough to cause a temperature rise of the melt, neither being able to directly cause ignition. However, the effect of oxidation on prolonging the solidification time of the castings cannot be ignored. As mentioned above, the experiment has confirmed that the critical ignition time of the AZ31 casting at 1073 K is not more than 10 min and the corresponding casting wall thickness is ∼5 cm. However, when considering the effect of heat released during oxidation, the solidification time of castings with wall thickness of 5 cm will be longer than 10 min, as shown in Figure 9. From the perspective of increased solidification time, the possibility of ignition becomes much higher.

#### 3.3.2 The thermal effects of magnesium vapor oxidation

After the rupture of surface oxide scale, the stable oxidation controlled by diffusion changes to the oxidation of magnesium vapor, due to the high vapor pressure of magnesium. Czerwinski et al. found that the evaporation rate of magnesium follows the Arrhenius relation (Czerwinski 2002):

$$K_{evap}=0.6\exp\left(\frac{-25000}{RT}\right)\;g/cm^{2}s$$ (17)

According to the heat released during oxidation described in Eq. 16, the relationship between the heat release of magnesium vapor and temperature can be expressed as follow:

$$\Delta H_{evap}=0.025\times \left[3.97582T\left(t\right)+857598.01099\right]\exp\left[\frac{-25000}{RT\left(t\right)}\right]\;J/cm^{2}s$$ (18)

Where the oxidation temperature *T* is a function of time *t*. The heat released after oxide rupture equals to the heat released from the melt cooling and magnesium vapor oxidation:

$$q=\Delta H_{S}\frac{\rho_{L}h}{m}\frac{\partial T}{\partial t}+\Delta H_{evap}$$ (19)

The temperature of the casting after oxide rupture can be induced from Eqs. 10, 19:

$$\Delta H_{S}\frac{\rho_{L}h}{m}\ln\left(\frac{T_{P}-T_{0}}{T_{S}-T_{0}}\right)=2\sqrt{\frac{\lambda \rho c}{\pi }}\sqrt{t}-\int_{0}^{t}\frac{\Delta H_{evap}\left(T_{S}\right)}{T_{S}-T_{0}}dt$$ (20)

**FIGURE 10:**
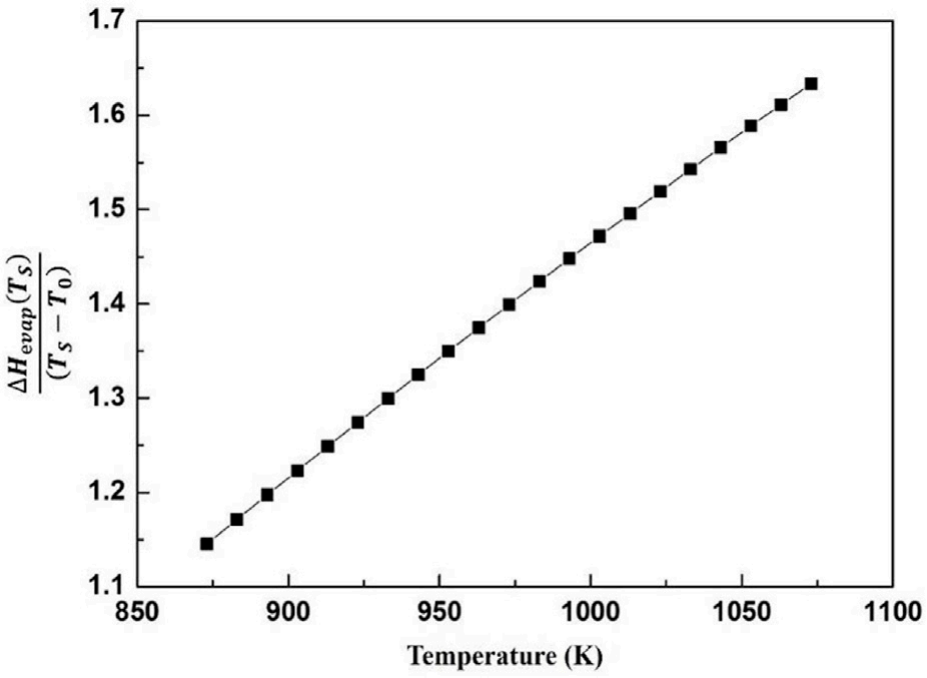
The heat release of magnesium vapor oxidation as a function of temperature.

Where the temperature of the casting *Ts* is a function of time *t* and the relationship between $\frac{\Delta H_{evap}\left(T_{S}\right)}{T_{S}-T_{0}}$ and temperature is calculated as shown in Figure 10. From the view of Figure 10, there is a linear relationship between $\frac{\Delta H_{evap}\left(T_{S}\right)}{T_{S}-T_{0}}$ and temperature, and the vapor rate changes to a lower degree in the temperature range of solidification. Hence, Eq. 20 is approximately estimated as Eq. 21,

$$\Delta H_{S}\frac{\rho_{L}h}{m}\ln\left(\frac{T_{P}-T_{0}}{T_{S}-T_{0}}\right)=2\sqrt{\frac{\lambda \rho c}{\pi }}\sqrt{t}-\frac{\Delta H_{evap}\left(T_{avg}\right)}{T_{avg}-T_{0}}t$$ (21)

Where *T*_*avg*_ is the average value of pouring temperature and solidus temperature.

**FIGURE 11:**
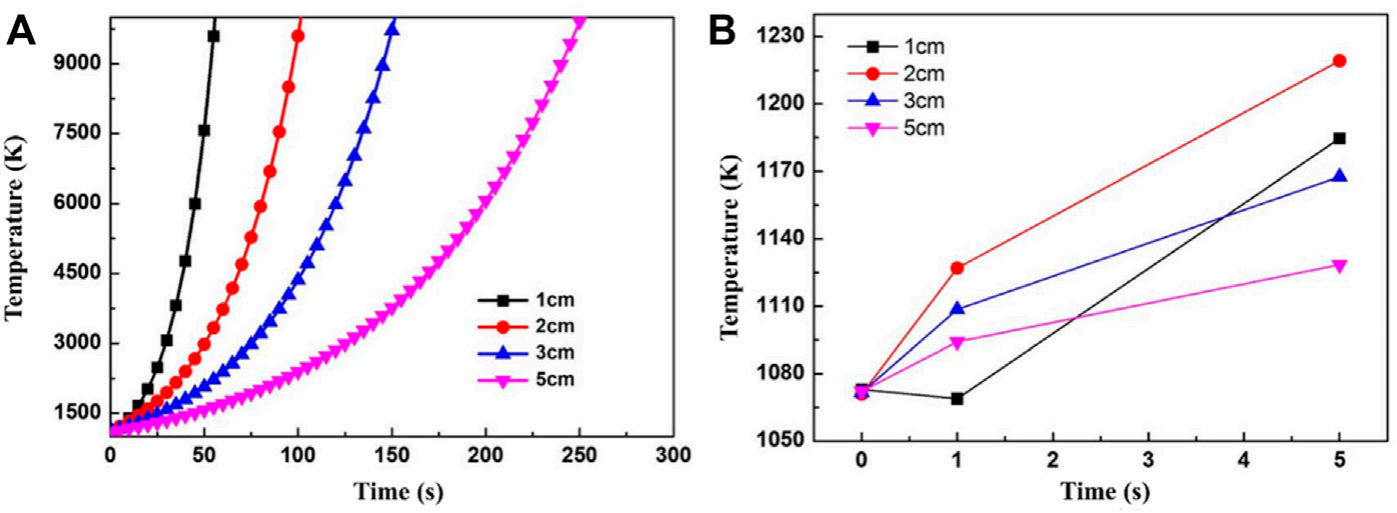
The effects of magnesium vapor oxidation on the cooling of castings. **(A)** from 0 to 300 min **(B)** from 0 to 5 min.

The effects of heat release from magnesium vapor oxidation on the cooling curve of the castings are calculated based on the equations above, as shown in Figure 11. The melt temperature will not decrease until the magnesium vapor begins to oxidize. It indicates that the heat release of oxidation of magnesium vapor is much greater than the heat dissipation capacity of the mold. Once magnesium vapor oxidation occurs, the temperature of the melt will gradually rise with time. In fact, a higher oxidation temperature induces more serious oxidation reaction and more heat release, and leads to the spontaneous combustion of the melt. Therefore, if the surface oxide layer breaks, the molten metal will inevitably ignite.

When the wall thickness of the casting is less than 1 cm, the melt temperature drops briefly at the initial stage of the oxide layer fracture, but rises quickly after 1 s, as shown in Figure 11B. This is due to the large temperature gradient in the mold at the beginning, and the mold maintains a good heat-transfer ability. But it is obviously not enough to complete the solidification of the casting. Therefore, even though the wall thickness of the casting is very small, the rupture of the oxide scale still could cause a temperature rise and combustion. It should be noticed that, in this section, it is approximately considered that the internal and external temperatures of the casting are uniform. Hence, greater wall thicknesses of the castings induce a slower rate of temperature increase. However, there is a certain temperature gradient between the surface and the interior of the actual casting. The surface temperature is low and the center temperature is high and the temperature rise of casting surface caused by the actual oxide scale rupture will be more pronounced.

According to the calculations above, the stable oxidation controlled by diffusion cannot cause melt ignition and the melt temperature almost immediately rises after the rupture of the surface oxide. It proves that the rupture of the surface oxide scale is the critical condition for melt ignition. Although diffusion-controlled oxidation cannot directly cause ignition of the castings, its effect on delaying the solidification time of molten magnesium alloys is still worth notice. The ignition point of magnesium alloy is near the solidus temperature and the alloy gains ignition risk during the solidification process. This is especially true for castings with large modulus, where stable oxidation heat release obviously increases the solidification time, thus brings more ignition risk. Therefore, a relationship between the casting modulus and ignition has been identified. If the casting exceeds the critical modulus under certain conditions, it may lead to spontaneously combustion. The critical modulus is related to the pouring temperature, which determines the oxide growth rate at the beginning of the casting process.

### 3.4 Critical conditions for surface oxide rupture during melt cooling

The rupture of the surface oxide scale is mainly attributed to stress in the oxide scale. During the casting processes, the melt and the surface oxide scale both shrink with a decrease in temperature. However, the thermal expansion coefficient of the metal is larger than that of the oxide and thermal stress in the surface oxide gradually increases during casting solidification. As shown in Figure 2, the wrinkle morphology of the surface oxide can be observed after the melt has undergone a relatively long-term oxidation. The thermal stress in oxide was investigated based on an infinite plate model by Evans, where the growth stress in the oxide was ignored (Evans 1995):

$$\sigma_{OX}=-\frac{E_{OX}\Delta T\left(\alpha_{m}-\alpha_{OX}\right)}{\left(1-\nu \right)\left(1+\frac{E_{OX}\xi }{E_{m}h}\right)}$$ (22)

Where *E*_*OX*_ is the Young’s modulus of the oxide, *α* is thermal expansion coefficient and *ν* is Poisson’s ratio. *ξ* and *h* are the thicknesses of the oxide scale and metal, respectively. As the thickness of the oxide is very small compared to the thickness of metal, it may be neglected. Equation 22 can thus be rewritten as:

$$\sigma_{OX}=-\frac{E_{OX}\Delta T\left(\alpha_{m}-\alpha_{OX}\right)}{\left(1-\nu \right)}$$ (23)

The critical temperature changes for buckling and wedging cracks can be estimated through following equations (Evans 2005):

$$\mathrm{Buckling}\;crack\mathrm{: \Delta}T=\frac{1.22}{\Delta \alpha \left(1-\nu_{OX}^{2}\right)}{\left(\frac{\xi }{r}\right)}^{2}$$ (24)

$$\mathrm{Wedging}\;crack\mathrm{: \Delta}T_{C}={\left[\frac{\gamma_{F}}{\xi E_{OX}{\left(\Delta \alpha \right)}^{2}\left(1-\nu_{OX}\right)}\right]}^{1/2}$$ (25)

Where Δ*α* is the thermal expansion difference of the oxide and metal, *ν*_*OX*_ is the Poisson’s ratio of the oxide and *r* is the buckling radius of the wrinkle region. *γ*_*F*_ is the energy per unit area required to produce a fresh surface at the oxide metal interface.

**FIGURE 12:**
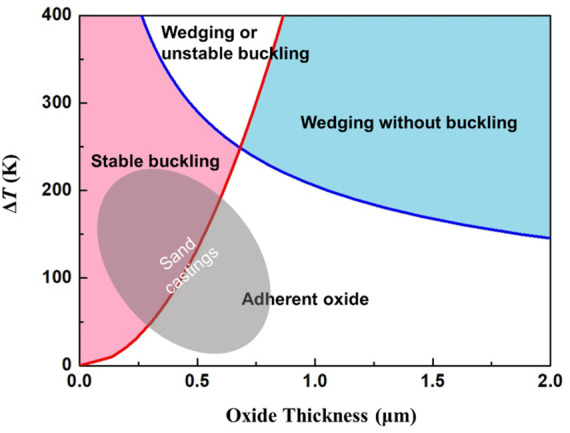
The spallation diagram for MgO on magnesium alloy.

Based on Eqss 24, 25, the spallation diagram for MgO on magnesium alloy can be obtained with the parameters listed in Table 1, as shown in Figure 12. This diagram describes the relevance of MgO rupture behaviors and its thickness during different temperature changes. There are two lines representing the limiting conditions for buckling and wedging, which intersect and divide the diagram into four domains. The line on the left-hand side of the diagram corresponds to the condition for stable buckling given by Equation 24. It should be noticed that the oxide thickness is assumed to be constant when calculating this diagram even though the oxide gradually grows in the cooling processes of castings. According to the experiment and calculation results shown in Figure 6 and Figure 7, the oxide mainly grows at high temperatures above the liquidus temperature of metal. During the solidification process, the oxide scale thickness at a much slower rate. The maximum oxide thickness and attained temperature drop during solidification of the sand-mold castings approximately distribute in the shadow region marked in Figure 12. Therefore, for the sand-mold casting processes, the surface oxide mainly cracks by routes of stable buckling.

**TABLE 1:** The parameters used for magnesium oxide spallation diagram calculation (Mitchell 1999).

Parameters	Thermal expansion coefficient difference K^−1^	Fracture energy J/m^2^	Young’s modulus GPa	Poisson’s ratio
Values	2.6 × 10^−5^	5	270	0.35

Figure 12 shows the critical temperature drop needed to initiate stable buckling and wedging cracks. However, the critical conditions for oxide crack during casting cannot be directly estimated based on Figure 12 due to the changing of oxide thickness with time. As it is proved that the oxide on the melt mainly cracks by routes of buckling, in order to find the underlying relevance of the temperature drop and oxide growth to buckling cracks, Eq. 24 is derived with time *t*.

$$\frac{dT}{dt}\frac{\Delta \alpha \left(1-\nu_{OX}^{2}\right)r^{2}}{2.44\xi }=\frac{d\xi }{dt}$$ (26)

**FIGURE 13:**
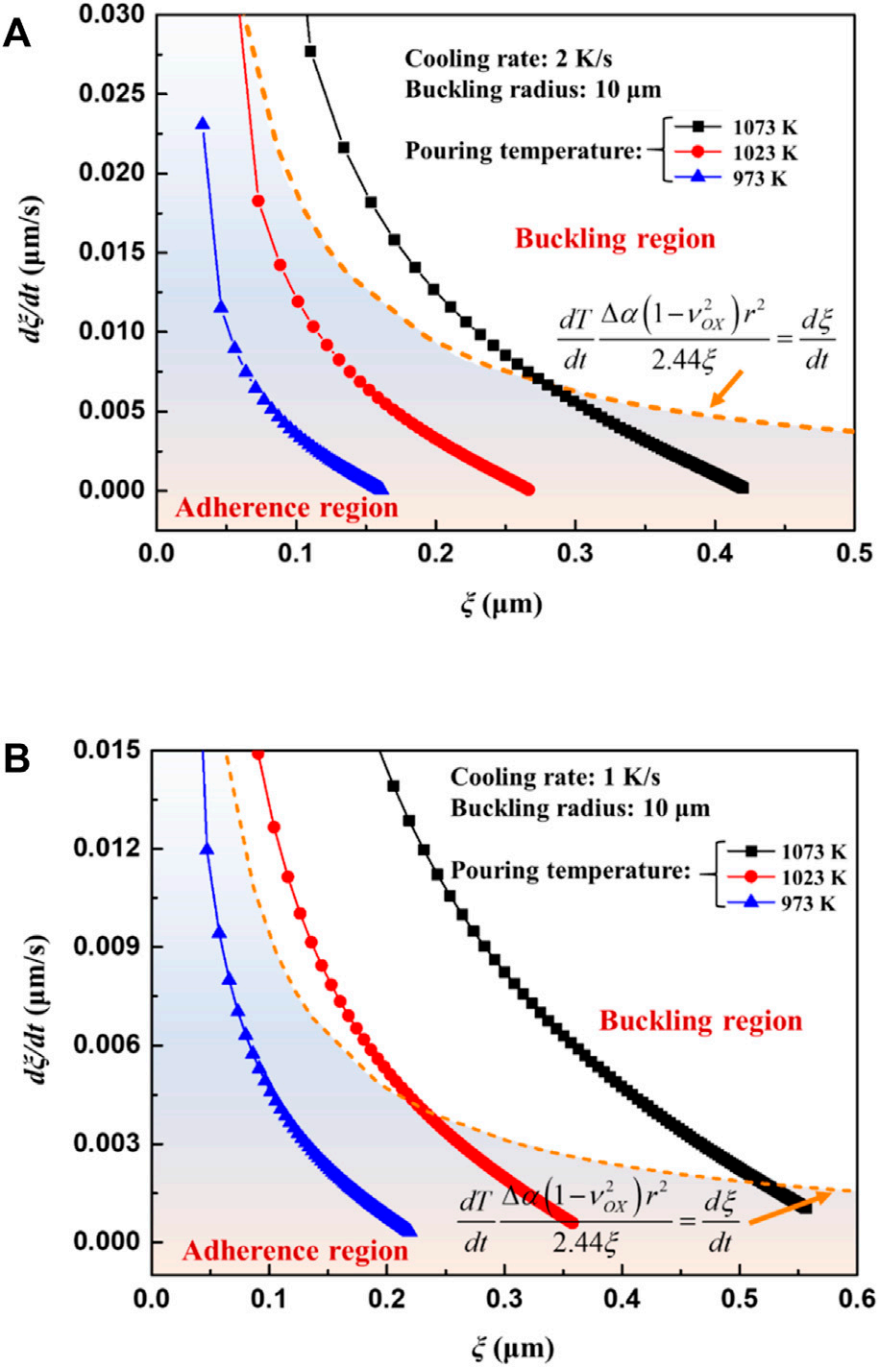
The oxide growth rate curves as a function of oxide thickness under different pouring temperatures **(A)** cooling rate 2 K/s **(B)** cooling rate 1 K/s.

Eq. 26 gives the critical oxide growth rate for buckling. When the oxide growth rate is larger than the critical value, buckling cracks initiate. Combined with the oxide growth calculation mentioned above in Section 3.2, the occurrence of buckling crack in different casting conditions can be estimated. Figure 13 illustrates the effects of pouring temperature on the buckling crack of the oxide at constant cooling rates. The region under the curve of Eq. 26 shows that no oxide cracks occur. For the castings with a cooling rate of 2 K/s, pouring at 1073 K may induce buckling crack of the oxide and ignition of the melt. Decreasing the pouring temperature to 1023 K can result in inhibition of ignition, as shown in Figure 13A. However, pouring at 1023 K still causes ignition when the cooling rate decreases to 1 K/s, as shown in Figure 11B.

It is still difficult to accurately estimate the oxide scale rupture by the oxide layer growth rate since it may not be experimentally verified, and neither a qualitative nor quantitative relationship between casting parameters and the growth rate of oxide can be clearly attained. According to Figure 13, it can be seen that oxide scale breaks during the cooling process are mainly determined by the pouring temperature and cooling rate. At the same pouring temperature, the casting tends to ignite when the cooling rate is reduced, and at the same cooling rate, increasing the pouring temperature will also cause the casting to ignite. These findings are consistent and concur with actual casting experiences. Moreover, there must be a critical cooling rate for ignition. When the pouring temperature is constant, if the cooling rate of the casting is lower than the critical cooling rate, the melt will burn, otherwise it will not. In order to directly establish the relationship between casting parameters and oxide scale rupture, a new ignition criterion relevant to pouring temperature and cooling rate is deduced by substituting the oxide layer growth rate Eq. 2 into Eq. 26,

$$\frac{dT}{dt}\frac{\Delta \alpha \left(1-v_{OX}^{2}\right)r^{2}}{2.44}>-V_{0}\Delta C_{M{\text{g}}^{2+}}D_{0}\exp\left(\frac{-Q}{RT}\right)$$ (27)

**FIGURE 14:**
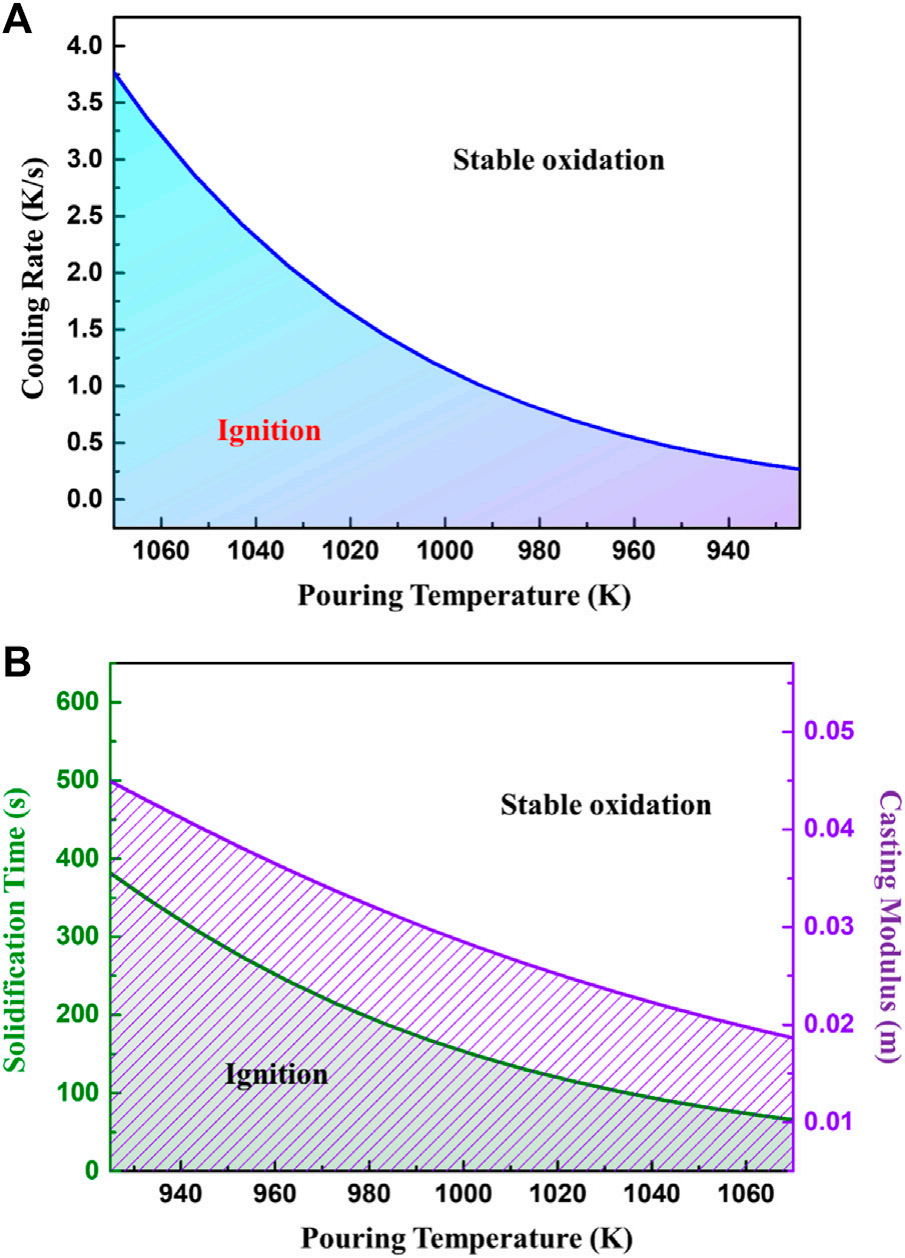
The ignition criteria for casting cooling processes. **(A)** critical cooling rate and **(B)** critical solidification time and casting modulus as a function of pouring temperature.

When it is plotted, the critical cooling rates varying with pouring temperatures are obtained as shown in Figure 14A. According to Figure 14A, the critical cooling rate increases exponentially with the rising of the pouring temperature. Therefore, it may be deduced that a high pouring temperature significantly increases the risk for ignition.

The modulus of casting is commonly used in the design of casting processes, which is the ratio of casting volume and its surface area. As the cooling rate is relevant to the solidification time and modulus of casting, the critical cooling rates can be converted to these two parameters, seen in Figure 14B. Obviously, with an increase of pouring temperature, the critical ignition modulus of the casting decreases and the allowable solidification time shortens. The relationship between critical ignition modulus and pouring temperature is approximately linear. When pouring between 973 and 1023 K calculated by MgO parameters, the corresponding critical modulus of the casting is between 33 and 25 mm. In these cases, the casting should solidify within 210 and 120 s respectively, to inhibit ignition during solidification.

## 4 Conclusion

In this paper, the oxidation behavior of AZ31 in different solidification conditions was studied. Based on the experimental and calculated results, the underlying relevance of ignition to casting process parameters was discussed. The main conclusions are summarized as follows:

(1) The oxide growth kinetics during solidification is successfully estimated based on Fick’s first law. According to the analysis of the heat release during the oxidation reaction, it is proved that stable oxidation could not induce ignition and that magnesium vapor oxidation caused by oxide rupture directly attributes to melt combustion.
(2) The surface oxide scale morphologies change from flat to wrinkles and finally cracks with a decrease in cooling rate. The spallation diagram for MgO is established based on oxide spallation models. Both experiments and calculations show the MgO scale cracks by a route of stable buckling.
(3) There is a critical cooling rate for melt ignition during casting, and it varies with pouring temperature. The quantified relationship between pouring temperature and critical cooling rate is determined and expressed. Commonly used casting process parameters like solidification time and casting modulus can also be employed to estimate the possibility of ignition.

## Data Availability

The original contributions presented in the study are included in the article/supplementary material, further inquiries can be directed to the corresponding author.
